# Crystal structures of three zinc(II) halide coordination complexes with quinoline *N*-oxide

**DOI:** 10.1107/S2056989022005953

**Published:** 2022-06-10

**Authors:** Clifford W. Padgett, Will E. Lynch, Erin N. Groneck, Melina Raymundo, Desiree Adams

**Affiliations:** a Georgia Southern University, 11935 Abercorn St., Department of Chemistry and Biochemistry, Savannah GA 31419, USA

**Keywords:** crystal structure, zinc(II) coordination complex, quinoline *N*-oxide, Hirshfeld surface analysis

## Abstract

The reaction of one equivalent of zinc(II) halide with two equivalents of quinoline *N*-oxide (QNO) in methanol yields compounds as Zn*X*_2_(QNO)_2_, where *X* = Cl (**I**), Br (**II**) and I (**III**), namely, di­chlorido­bis­(quinoline *N*-oxide-κ*O*)zinc(II), [ZnCl_2_(C_9_H_7_NO)_2_], di­bromido­bis­(quinoline *N*-oxide-κ*O*)zinc(II), [ZnBr_2_(C_9_H_7_NO)_2_], and di­iodido­bis­(quinoline *N*-oxide-κ*O*)zinc(II) [ZnI_2_(C_9_H_7_NO)_2_]. In all three complexes, Zn cations are coordinated by two QNO ligands bound through the oxygen atoms and two halide atoms, with *X*—Zn—*X* bond angles *ca* 20° wider than the O—Zn—O, giving rise to a distorted tetra­hedral geometry. Crystals of (**II**) and (**III**) are isostructural and both show pairwise π-stacking of QNO ligands and weak C—H⋯*X* hydrogen bonds, while (**I**) packs differently, with a shorter C—H⋯Cl bond and without π-stacking.

## Abstract 2

The structures of the three related compounds di­chlorido­bis­(quinoline *N*-oxide-κ*O*)zinc(II); di­bromido­bis­(quinoline *N*-oxide-κ*O*)zinc(II) and di­iodido­bis­(quinoline *N*-oxide-κ*O*)zinc(II) are presented.

## 1. Chemical context

*N*-oxide complexes have a rich history in organic transformations, including utility with transition metals in oxotransformations [see, for example, Eppenson (2003 ▸) and Moustafa *et al.* (2014 ▸)]. These transition-metal *N*-oxide complexes highlight the strong Lewis acid/Lewis base properties of the zinc(II) ion and *N*-oxides, respectively. Aromatic *N*-oxides are strong Lewis base ligands and form transition-metal complexes that are prevalent in the literature and highlight the strong transition metal inter­actions with the lone pair on the *N*-oxide oxygen atom. Examples of such complexes include a 4-methyl­pyridine *N*-oxide (MePyNO) derivative CuCl_2_·2MePyNO (CMPYUC; Watson & Johnson, 1971 ▸) and pyridine *N*-oxide (6PyNO) derivatives Ni(BF_4_)_2_·6PyNO (PYNONI; van Ingen Schenau *et al.*, 1974 ▸) or Au(CF_3_)_3_·PyNO (NEPVOW; Pérez-Bitrián *et al.*, 2017 ▸). Previous reports of zinc(II) complexes with aromatic *N*-oxides include di­bromo­bis­(4-meth­oxy­pyridine *N*-oxide-κ*O*)zinc(II) (GAWHIW; Shi *et al.* 2005*a* ▸), di­aqua­bis­(picolinato *N*-oxide-κ^2^ *O*,*O*′)zinc(II) (XISBOR; Li *et al.*, 2008 ▸) and di­chloro­bis­(pyridine *N*-oxide)zinc(II) (QQQBXP01; McConnell *et al.*, 1986 ▸), all of which are mononuclear complexes.

Herein we report the crystal structures of three complexes of quinoline *N*-oxide (QNO) with zinc(II) chloride, bromide and iodide. All three were obtained by 1:2 stoichiometric reaction of the zinc(II) halide with QNO in methanol and found to be mononuclear Zn*X*_2_(QNO)_2_ complexes with a distorted tetra­hedral environment around the zinc ion. 
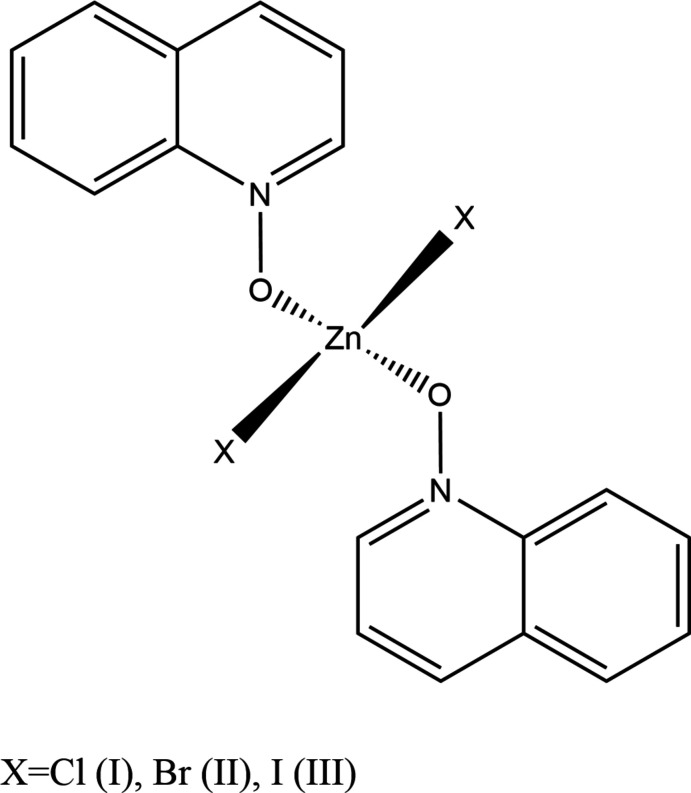


## 2. Structural commentary

**Figure 1:**
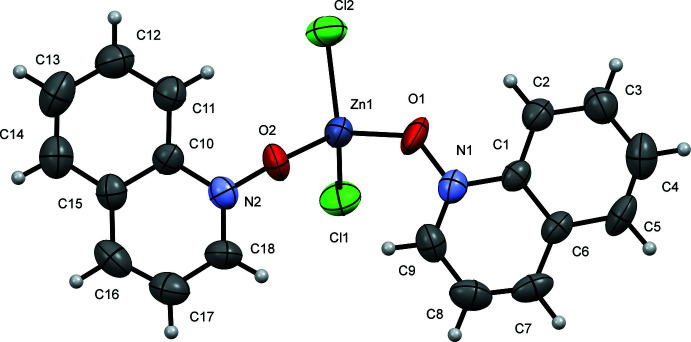
A view of compound (**I**), showing the atom labeling. Displacement ellipsoids are drawn at the 50% probability level.

**Figure 2:**
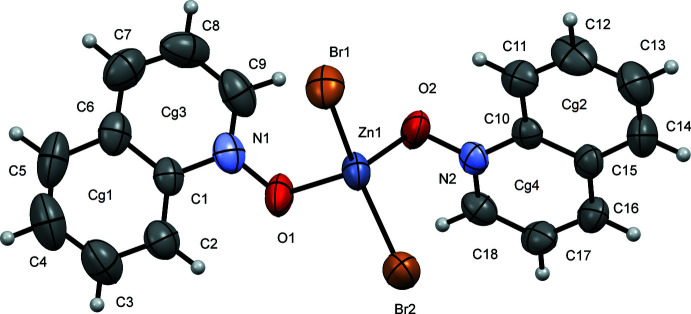
A view of compound (**II**), showing the atom labeling. Displacement ellipsoids are drawn at the 50% probability level.

**Figure 3:**
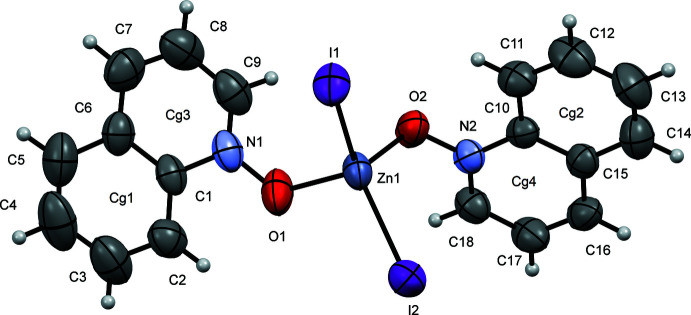
A view of compound (**III**), showing the atom labeling. Displacement ellipsoids are drawn at the 50% probability level.

**Figure 4:**
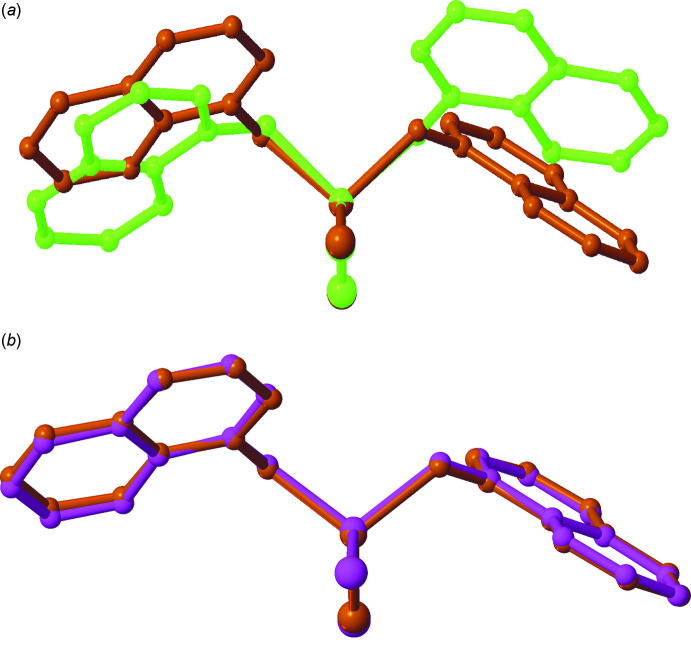
(*a*) Mol­ecular overlay of compound (**I**) (green) and compound (**II**) (brown). (*b*) Mol­ecular overlay of compound (**II**) (brown) and compound (**III**) (purple).

Compound (**I**) crystallizes in the monoclinic space group *P*2_1_ (Fig. 1 ▸), whereas compounds (**II**) (Fig. 2 ▸) and (**III**) (Fig. 3 ▸) both crystallize in the monoclinic space group *P*2_1_/*c*. Each structure contains one symmetrically independent mol­ecule, the coordination sphere around each Zn atom being a distorted tetra­hedron. Selected bond lengths and angles in these complexes are shown in Table 1 ▸. Compounds (**II**) and (**III**) are isostructural in both the mol­ecular conformation and crystal packing, while (**I**) differs in both aspects, as illustrated by an overlay of mol­ecules (**I**) and (**II**) (Fig. 4 ▸ *a*) on one hand, and mol­ecules (**II**) and (**III**) on the other (Fig. 4 ▸ *b*). Most notably, (**I**) differs in the orientation of the QNO rings relative to each other, the C2—N1—N2—C11 torsion angles being −16.9 (5)° in (**I**) *versus* −113.9 (3)° in (**II**) and −111.6 (3)° in (**III**).

## 3. Supra­molecular features

**Figure 5:**
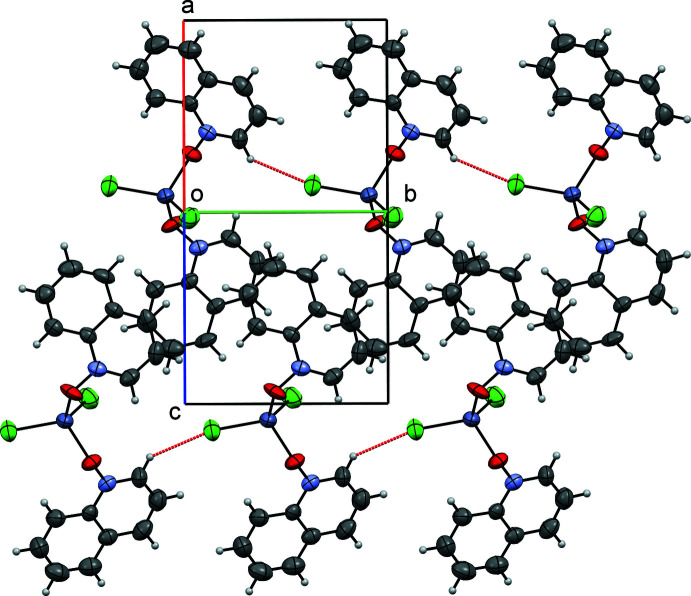
Crystal packing diagram of compound (**I**), viewed down the [101] direction.

**Figure 6:**
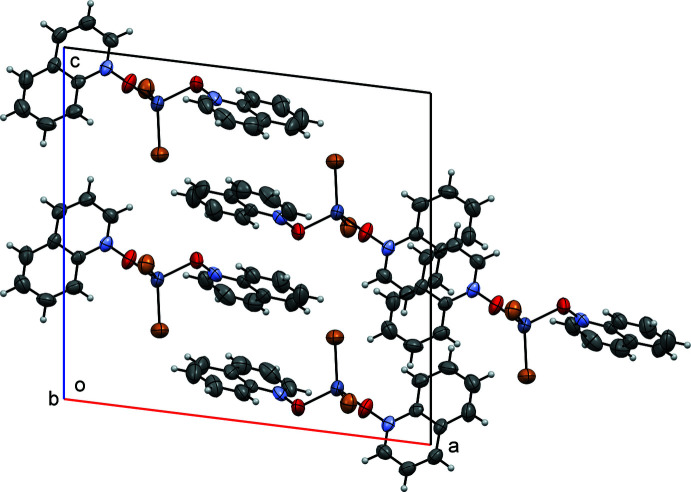
Crystal packing diagram of compound (**II**), viewed down the *b* axis.

Figs. 5 ▸, 6 ▸ and 7 ▸ show the packing of compounds (**I**), (**II**) and (**III**), respectively. In the crystal structures, the packing is stabilized by van der Waals inter­actions and, in (**II**) and (**III**), by similar systems of pairwise π–π stacking inter­actions. Quinoline moieties *Cg*1–*Cg*3 and *Cg*2–*Cg*4 (see Figs. 6 ▸ and 7 ▸) are stacked each against its own inversion-related equivalent, with the separations between their (parallel) mean planes equaling 3.483 (5) and 3.402 (5) Å, respectively, for (**II**), 3.466 (5) and 3.436 (5) Å for (**III**). The structure of (**I**) has no π-stacking. Besides, all three structures are characterized by C—H⋯*X* hydrogen bonds (*X* = halogen), see below.

## 4. Hirshfeld surface analysis

**Figure 8:**
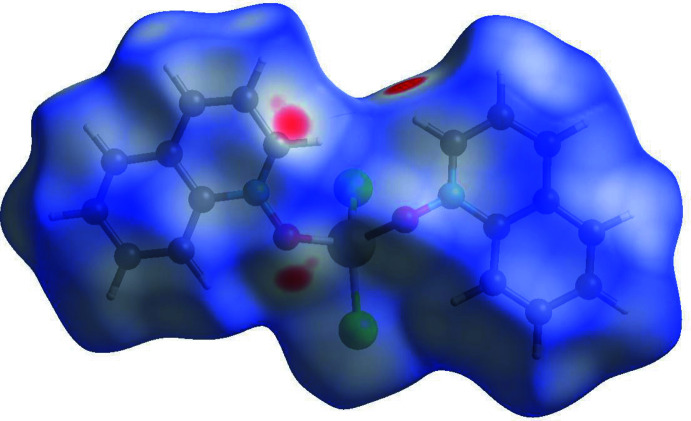
Hirshfeld surface for (**I**) mapped over *d*_norm_.

The inter­molecular inter­actions were further investigated by qu­anti­tative analysis of the Hirshfeld surface, and visualized with *Crystal Explorer 21* (Spackman *et al.*, 2021 ▸) and the two-dimensional fingerprint plots (McKinnon *et al.*, 2007 ▸). Figs. 8 ▸, 9 ▸ and 10 ▸ show Hirshfeld surfaces of mol­ecules (**I**) to (**III**) mapped with the function *d*_norm_, the sum of the distances from a surface point to the nearest inter­ior (*d*_i_) and exterior (*d*_e_) atoms, normalized by the van der Waals (vdW) radii of the corres­ponding atoms (r_vdW_). Contacts shorter than the sums of vdW radii are shown in red, those longer in blue, and those approximately equal to vdW as white spots.

For (**I**), the most intense red spots correspond to the inter­molecular contacts O1⋯C9(1 − *x*, *y* − 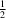 , 1 − *z*) [3.048 (9) Å] and the hydrogen bond C18—H18⋯Cl2(*x*, *y* + 1, *z*). The latter has the distances H⋯Cl = 2.53 Å (for the C—H distance normalized to 1.083 Å) and C⋯Cl = 3.416 (9) Å within the previously observed range but shorter than the average values of 2.64 and 3.66 Å, respectively (Steiner, 1998 ▸). The other chloride ligand, Cl2, forms four H⋯Cl contacts of 2.83–2.98 Å, more typical for van der Waals inter­actions (Rowland & Taylor, 1996 ▸). For (**II**) and (**III**), the red spots correspond to C—H⋯*X* inter­actions, *viz*. C18—H18⋯*X*1, C5—H5⋯*X*1, C16—H16⋯*X*2, and C9—H9⋯*X*2, which can be also regarded as weak hydrogen bonds (Steiner, 1998 ▸). The H⋯*X* distances in (**II**) (*X* = Br) are 2.85, 2.88, 2.88 and 2.89 Å, respectively, while in (**III**) (*X* = I) they are 3.03, 3.12, 3.03 and 2.96 Å, respectively.

Analysis of the two-dimensional fingerprint plots (Table 2 ▸) indicates that H⋯H contacts are the most common in all three structures. *X*⋯H contacts make the second highest contribution, which increases in the succession (**I**) < (**II**) < (**III**), together with the size of the halogen atoms and hence their share of the mol­ecular surface (16.9, 18.5 and 20.6%, respectively). Inter­estingly, π-stacking in the structures of (**II**) and (**III**) gives only a modest increase of C⋯C contacts compared to (**I**), probably because it is counterbalanced by an overall decrease of carbon atoms’ share of the surface (21.4 > 19.5 > 18.3%). No halogen⋯halogen contacts are observed in any of the three structures.

## 5. Database survey

A search in the Cambridge Structural Database (CSD, version 5.42, update of February 2021; Groom *et al.*, 2016 ▸) for aromatic *N*-oxides and halogen ligands bound to zinc returned 21 unique entries, the majority (15) of which contain pyridine *N*-oxide and its derivatives. Of these, the most closely related are pyridine *N*-oxide complexes, di­chloro­bis­(pyridine *N*-oxide)zinc(II) (QQQBXP01; McConnell *et al.*, 1986 ▸), di­bromo­robis(pyridine *N*-oxide)zinc(II) (FIPVUV; Edwards *et al.*, 1999 ▸) and di­iodo­robis(pyridine *N*-oxide)zinc(II) (IPNOZN01; Edwards *et al.*, 1999 ▸). Related to these are methyl derivatives of pyridine *N*-oxide complexes with ZnCl_2_, *viz*. di­chloro­bis­(2,6-di­methyl­pyridine *N*-oxide)zinc(II) (LUTOZN; Sager & Watson, 1968 ▸), three isomers of di­chloro­bis­(methyl­pyridine *N*-oxide)zinc(II) (QQQBXG, QQQBXJ, QQQBXM), for which only unit-cell parameters were determined (Kidd *et al.*, 1967 ▸), and finally, di­iodo­bis­(4-methyl­pyridine *N*-oxide)zinc(II) (SANRUV; Shi *et al.*, 2005*b* ▸). There is one known structure of a quinoline *N*-oxide derivative, di­chloro­bis­(2-methyl­quinoline *N*-oxide)zinc(II) (AFUSEZ; Ivashevskaja *et al.*, 2002 ▸).

## 6. Synthesis and crystallization

The water content of QNO and ZnBr_2_ have been determined by Thermal Gravimetric Analysis. The formulation for each was found to be QNO·0.28H_2_O (*M*_W_ = 150.21 g mol^−1^) and ZnBr_2_·0.86H_2_O (*F*_W_ = 240.69 g mol^−1^).

The title compounds were all synthesized in a similar manner. Compound (**I**) was synthesized by dissolving 0.0986 g of QNO·0.28H_2_O (0.656 mmol, purchased from Aldrich) in 33 mL of methanol to which 0.0440 g of ZnCl_2_ (0.176 mmol, purchased from Strem Chemicals) were added at 295 K. The solution was covered with parafilm then allowed to sit; X-ray quality crystals were grown by slow evaporation at 295 K. Yield, 0.0822 g (60.2%). Selected IR bands (ATR–IR, cm^−1^): 3107 (*w*), 3083 (*w*), 3057 (*w*), 1579 (*m*), 1513 (*m*), 1447 (*m*), 1402 (*s*), 1269 (*s*), 1227 (*m*), 1203 (*s*), 1179 (*m*), 1144 (*m*), 1089 (*s*), 1050 (*m*), 883 (*s*), 800 (*s*), 768 (*s*), 723 (*m*), 584 (*m*), 559 (*m*), 542 (*m*).

Compound (**II**) was synthesized by dissolving 0.0983 g of QNO·0.28H_2_O (0.654 mmol), in 40 mL of methanol to which 0.0778 g of ZnBr_2_·0.86H_2_O (0.323 mmol, purchased from Alfa Aesar) were added at 295 K. The solution was covered with parafilm then allowed to sit; X-ray quality crystals were grown by slow evaporation at 295 K. Yield, 0.0866 g (46.7%). Selected IR bands (ATR–IR, cm^−1^): 3106 (*w*), 3075 (*w*), 3061 (*w*), 3016 (*w*), 1580 (*m*), 1510 (*s*), 1455 (*m*), 1270 (*s*), 1227 (*m*), 1214 (*s*), 1204 (*s*), 1173 (*m*), 1138 (*m*), 1086 (*s*), 1048 (*m*), 877 (*m*), 800 (*s*), 767 (*s*), 720 (*s*), 581 (*m*), 563 (*m*), 500 (*m*).

Compound (**III**) was synthesized by dissolving 0.0517 g of QNO·0.28H_2_O (0.352 mmol) in approximately 36 mL of methanol to which 0.0524 g of ZnI_2_ (0.164 mmol, purchased from Aldrich) were added at 295 K. The solution was covered with parafilm then allowed to sit; X-ray quality crystals were grown by slow evaporation at 295 K. Yield, 0.0910 g (52.3%). Selected IR Bands (ATR–IR, cm^−1^): 3100 (*w*), 3090 (*w*), 2076 (*w*), 3059 (*w*), 3027 (*w*),1580 (*s*), 1507 (*s*), 1382 (*s*), 1267 (*m*), 1225 (*m*), 1207 (*s*), 1169 (*m*), 1141 (*m*), 1044 (*m*), 880 (*s*), 807 (*s*), 769 (*s*), 720 (*m*), 580 (*m*), 562 (*m*), 499 (*m*).

Infrared spectroscopy confirms the presence of the QNO ligand in all three complexes. Characteristic IR bands include weak νC—H aromatic stretches observed from 3020–3107 cm^−1^ and νN—O stretches of the bound *N*-oxide in the range 1350–1150 cm^−1^; notably, a medium band observed in the ligand at 1311 cm^−1^, appears at between 1225–1227 cm^−1^ in the three metal complexes. Finally, a broad absorbance in the free ligand from 3100–3500 cm^−1^ (assigned to the water νO—H stretch) is absent in all of the metal complexes (Mautner *et al.*, 2016 ▸).

## 7. Refinement

Crystal data, data collection and structure refinement details are summarized in Table 3 ▸. All carbon-bound H atoms were positioned geometrically and refined as riding: C—H = 0.95–0.98 Å with *U*_iso_(H) = 1.2*U*_eq_(C).

## Supplementary Material

Crystal structure: contains datablock(s) I, II, III. DOI: 10.1107/S2056989022005953/zv2014sup1.cif

Structure factors: contains datablock(s) I. DOI: 10.1107/S2056989022005953/zv2014Isup2.hkl

Structure factors: contains datablock(s) II. DOI: 10.1107/S2056989022005953/zv2014IIsup3.hkl

Structure factors: contains datablock(s) III. DOI: 10.1107/S2056989022005953/zv2014IIIsup4.hkl

CCDC references: 2176715, 2176714, 2176713

Additional supporting information:  crystallographic information; 3D view; checkCIF report

## Figures and Tables

**Figure 7:**
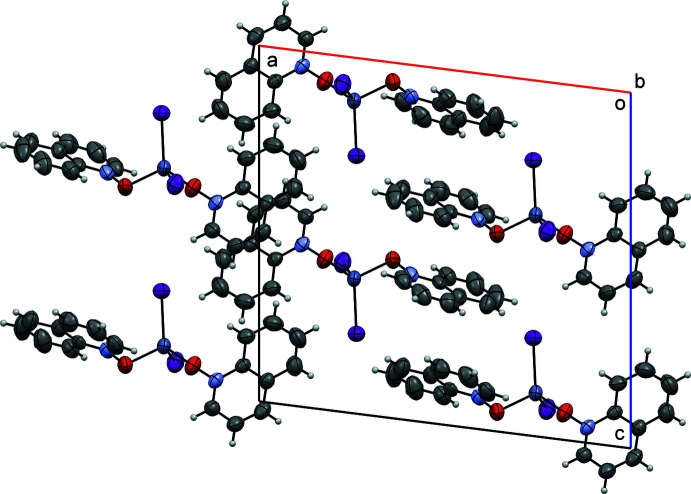
Crystal packing diagram of compound (**III**), viewed down the *b* axis.

**Figure 9:**
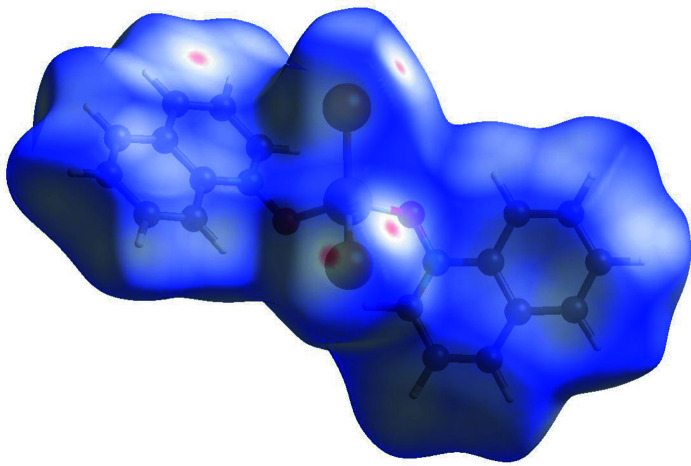
Hirshfeld surface for (**II**) mapped over *d*_norm_.

**Figure 10:**
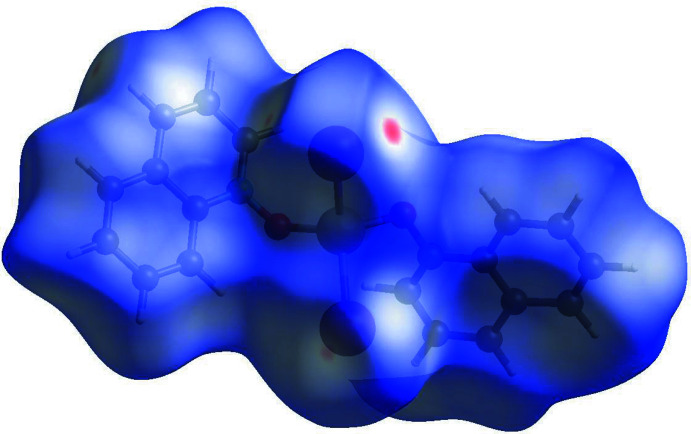
Hirshfeld surface for (**III**) mapped over *d*_norm_.

**Table 1:** Selected bond lengths and angles (Å, °)

Compound (**I**)		Compound (**II**)		Compound (**III**)	
Zn1—Cl1	2.215 (2)	Zn1—Br1	2.3575 (9)	Zn1—I1	2.5534 (8)
Zn1—Cl2	2.211 (2)	Zn1—Br2	2.3472 (10)	Zn1—I2	2.5475 (9)
Zn1—O1	1.991 (5)	Zn1—O1	1.975 (4)	Zn1—O1	1.974 (4)
Zn1—O2	1.959 (5)	Zn1—O2	1.989 (4)	Zn1—O2	1.995 (4)
Cl1—Zn1—Cl2	117.80 (9)	Br1—Zn1—Br2	123.45 (4)	I1—Zn1—I2	122.34 (3)
O1—Zn1—O2	99.4 (2)	O1—Zn1—O2	103.10 (16)	O1—Zn1—O2	104.12 (19)

**Table 2:** Contributions of selected inter­molecular contacts (%)

Compound	(**I**)	(**II**)	(**III**)
H⋯H	32.0	36.7	36.5
H⋯*X*/*X*⋯H	24.4	28.4	30.0
C⋯H/H⋯C	22.7	18.5	18.0
C⋯C	5.4	7.1	6.4
O⋯H/H⋯O	6.0	4.0	3.7

**Table 3:** Experimental details

	(**I**)	(**II**)	(**III**)
Crystal data			
Chemical formula	[ZnCl_2_(C_9_H_7_NO)_2_]	[ZnBr_2_(C_9_H_7_NO)_2_]	[ZnI_2_(C_9_H_7_NO)_2_]
*M*_r_	426.58	515.50	609.48
Crystal system, space group	Monoclinic, *P*2_1_	Monoclinic, *P*2_1_/*c*	Monoclinic, *P*2_1_/*c*
Temperature (K)	298	298	297
*a*, *b*, *c* (Å)	8.5167 (4), 7.8697 (4), 13.1617 (7)	16.3922 (11), 7.3527 (6), 15.5809 (10)	16.7231 (7), 7.6155 (4), 15.8689 (7)
β (°)	94.890 (5)	97.113 (6)	97.192 (4)
*V* (Å^3^)	878.94 (8)	1863.5 (2)	2005.08 (16)
*Z*	2	4	4
Radiation type	Mo *K*α	Mo *K*α	Mo *K*α
μ (mm^−1^)	1.72	5.62	4.32
Crystal size (mm)	0.1 × 0.1 × 0.03	0.15 × 0.08 × 0.03	0.3 × 0.3 × 0.3

Data collection			
Diffractometer	Rigaku XtaLAB mini	XtaLAB Mini (ROW)	Rigaku XtaLAB mini
Absorption correction	Multi-scan (*CrysAlis PRO*; Rigaku OD, 2019 ▸)	Multi-scan (*CrysAlis PRO*; Rigaku OD, 2019 ▸)	Multi-scan (*CrysAlis PRO*; Rigaku OD, 2019 ▸)
*T*_min_, *T*_max_	0.968, 1.000	0.833, 1.000	0.896, 1.000
No. of measured, independent and observed [*I* > 2σ(*I*)] reflections	5308, 3169, 2456	7207, 3415, 2095	11510, 3668, 2748
*R*_int_	0.036	0.043	0.032
(sin θ/λ)_max_ (Å^−1^)	0.602	0.602	0.602

Refinement			
*R*[*F* ^2^ > 2σ(*F* ^2^)], *wR*(*F* ^2^), *S*	0.044, 0.077, 1.03	0.042, 0.090, 1.02	0.035, 0.085, 1.07
No. of reflections	3169	3415	3668
No. of parameters	226	226	227
No. of restraints	1	0	0
H-atom treatment	H-atom parameters constrained	H-atom parameters constrained	H-atom parameters constrained
Δρ_max_, Δρ_min_ (e Å^−3^)	0.42, −0.35	0.55, −0.35	0.80, −0.81
Absolute structure	Flack *x* determined using 810 quotients [(*I* ^+^)−(*I* ^−^)]/[(*I* ^+^)+(*I* ^−^)] (Parsons *et al.*, 2013 ▸).	–	–
Absolute structure parameter	−0.006 (15)	–	–

Computer programs: *CrysAlis PRO* (Rigaku OD, 2019 ▸), *SHELXT2014/5* (Sheldrick, 2015*a* ▸), *SHELXL2018/1* (Sheldrick, 2015*b* ▸), and *OLEX2* (Dolomanov *et al.*, 2009 ▸).

## supplementary crystallographic information

### Dichloridobis(quinoline N-oxide-κO)zinc(II) (I) Crystal data

**Table:**

[ZnCl_2_(C_9_H_7_NO)_2_]	*F*(000) = 432
*M**_r_* = 426.58	*D*_x_ = 1.612 Mg m^−^^3^
Monoclinic, *P*2_1_	Mo *K*α radiation, λ = 0.71073 Å
*a* = 8.5167 (4) Å	Cell parameters from 1644 reflections
*b* = 7.8697 (4) Å	θ = 2.4–22.4°
*c* = 13.1617 (7) Å	µ = 1.72 mm^−^^1^
β = 94.890 (5)°	*T* = 298 K
*V* = 878.94 (8) Å^3^	Cube, clear colourless
*Z* = 2	0.1 × 0.1 × 0.03 mm

### Dichloridobis(quinoline N-oxide-κO)zinc(II) (I) Data collection

**Table:**

Rigaku XtaLAB mini diffractometer	3169 independent reflections
Radiation source: fine-focus sealed X-ray tube, Rigaku (Mo) X-ray Source	2456 reflections with *I* > 2σ(*I*)
Graphite monochromator	*R*_int_ = 0.036
ω scans	θ_max_ = 25.4°, θ_min_ = 2.4°
Absorption correction: multi-scan (CrysAlisPro; Rigaku OD, 2019)	*h* = −10→10
*T*_min_ = 0.968, *T*_max_ = 1.000	*k* = −9→9
5308 measured reflections	*l* = −15→14

### Dichloridobis(quinoline N-oxide-κO)zinc(II) (I) Refinement

**Table:**

Refinement on *F*^2^	Hydrogen site location: inferred from neighbouring sites
Least-squares matrix: full	H-atom parameters constrained
*R*[*F*^2^ > 2σ(*F*^2^)] = 0.044	*w* = 1/[σ^2^(*F*_o_^2^) + (0.0183*P*)^2^] where *P* = (*F*_o_^2^ + 2*F*_c_^2^)/3
*wR*(*F*^2^) = 0.077	(Δ/σ)_max_ < 0.001
*S* = 1.03	Δρ_max_ = 0.42 e Å^−^^3^
3169 reflections	Δρ_min_ = −0.35 e Å^−^^3^
226 parameters	Absolute structure: Flack *x* determined using 810 quotients [(*I*^+^)-(*I*^-^)]/[(*I*^+^)+(*I*^-^)] (Parsons *et al.*, 2013).
1 restraint	Absolute structure parameter: −0.006 (15)
Primary atom site location: dual	

### Dichloridobis(quinoline N-oxide-κO)zinc(II) (I) Special details

**Table:**

Geometry. All esds (except the esd in the dihedral angle between two l.s. planes) are estimated using the full covariance matrix. The cell esds are taken into account individually in the estimation of esds in distances, angles and torsion angles; correlations between esds in cell parameters are only used when they are defined by crystal symmetry. An approximate (isotropic) treatment of cell esds is used for estimating esds involving l.s. planes.

### Dichloridobis(quinoline N-oxide-κO)zinc(II) (I) Fractional atomic coordinates and isotropic or equivalent isotropic displacement parameters (Å^2^)

**Table:**

	*x*	*y*	*z*	*U*_iso_*/*U*_eq_	
Zn1	0.60832 (9)	0.40878 (9)	0.69325 (6)	0.0481 (2)	
Cl1	0.8131 (2)	0.5260 (3)	0.78185 (18)	0.0717 (6)	
Cl2	0.5724 (2)	0.1322 (2)	0.71012 (17)	0.0668 (6)	
O1	0.6087 (5)	0.4435 (7)	0.5459 (4)	0.0660 (17)	
O2	0.4152 (6)	0.5393 (6)	0.7184 (4)	0.0543 (13)	
N1	0.6919 (7)	0.5702 (8)	0.5068 (4)	0.0472 (15)	
N2	0.3927 (6)	0.6163 (7)	0.8067 (4)	0.0461 (14)	
C1	0.7938 (8)	0.5254 (9)	0.4342 (5)	0.0418 (17)	
C2	0.8061 (9)	0.3562 (9)	0.4045 (6)	0.052 (2)	
H2	0.745778	0.272501	0.432433	0.063*	
C3	0.9086 (10)	0.3150 (11)	0.3332 (6)	0.065 (2)	
H3	0.916168	0.203042	0.311636	0.077*	
C4	1.0011 (10)	0.4398 (14)	0.2932 (6)	0.071 (3)	
H4	1.072298	0.409606	0.246598	0.085*	
C5	0.9891 (9)	0.6041 (11)	0.3210 (6)	0.061 (2)	
H5	1.051065	0.685676	0.292553	0.074*	
C6	0.8835 (8)	0.6538 (9)	0.3931 (5)	0.0469 (18)	
C7	0.8623 (9)	0.8234 (8)	0.4243 (6)	0.056 (2)	
H7	0.920737	0.910057	0.397750	0.067*	
C8	0.7577 (10)	0.8601 (9)	0.4927 (6)	0.063 (2)	
H8	0.742052	0.972081	0.511924	0.075*	
C9	0.6733 (9)	0.7293 (10)	0.5342 (6)	0.056 (2)	
H9	0.602718	0.754824	0.582143	0.068*	
C10	0.3113 (8)	0.5307 (9)	0.8777 (6)	0.0441 (18)	
C11	0.2654 (9)	0.3621 (9)	0.8595 (6)	0.059 (2)	
H11	0.289239	0.306487	0.800371	0.071*	
C12	0.1846 (10)	0.2810 (12)	0.9306 (7)	0.073 (2)	
H12	0.154890	0.168062	0.920740	0.087*	
C13	0.1458 (11)	0.3686 (13)	1.0195 (7)	0.081 (3)	
H13	0.089040	0.312853	1.066778	0.097*	
C14	0.1899 (10)	0.5309 (12)	1.0360 (7)	0.069 (3)	
H14	0.163818	0.585631	1.094912	0.082*	
C15	0.2745 (8)	0.6187 (10)	0.9661 (5)	0.0508 (19)	
C16	0.3245 (9)	0.7899 (11)	0.9803 (6)	0.065 (2)	
H16	0.300458	0.850485	1.037580	0.078*	
C17	0.4081 (9)	0.8637 (10)	0.9084 (6)	0.067 (2)	
H17	0.443132	0.975085	0.917171	0.081*	
C18	0.4411 (9)	0.7745 (11)	0.8231 (6)	0.061 (2)	
H18	0.499384	0.826957	0.775384	0.073*	

### Dichloridobis(quinoline N-oxide-κO)zinc(II) (I) Atomic displacement parameters (Å^2^)

**Table:**

	*U*^11^	*U*^22^	*U*^33^	*U*^12^	*U*^13^	*U*^23^
Zn1	0.0494 (4)	0.0451 (5)	0.0519 (5)	−0.0013 (5)	0.0168 (4)	0.0023 (5)
Cl1	0.0656 (13)	0.0678 (14)	0.0812 (16)	−0.0173 (11)	0.0034 (12)	−0.0053 (12)
Cl2	0.0710 (14)	0.0421 (11)	0.0875 (16)	−0.0034 (10)	0.0087 (12)	0.0054 (10)
O1	0.066 (3)	0.083 (5)	0.052 (3)	−0.033 (3)	0.025 (3)	0.006 (3)
O2	0.059 (3)	0.063 (3)	0.043 (3)	0.011 (3)	0.017 (3)	−0.010 (3)
N1	0.045 (3)	0.057 (4)	0.040 (4)	−0.004 (3)	0.006 (3)	0.002 (3)
N2	0.042 (3)	0.050 (4)	0.046 (4)	0.007 (3)	0.004 (3)	−0.003 (3)
C1	0.041 (4)	0.047 (4)	0.037 (4)	−0.003 (4)	0.000 (3)	0.010 (4)
C2	0.052 (5)	0.056 (5)	0.048 (5)	−0.008 (4)	0.005 (4)	0.003 (3)
C3	0.071 (6)	0.064 (6)	0.060 (5)	0.005 (5)	0.012 (5)	−0.003 (4)
C4	0.065 (5)	0.097 (8)	0.053 (5)	0.012 (6)	0.016 (4)	0.008 (6)
C5	0.047 (5)	0.078 (6)	0.061 (6)	−0.006 (5)	0.018 (4)	0.027 (5)
C6	0.044 (4)	0.052 (5)	0.045 (4)	−0.008 (4)	0.004 (4)	0.010 (4)
C7	0.058 (5)	0.043 (5)	0.062 (5)	−0.012 (4)	−0.013 (4)	0.019 (4)
C8	0.076 (6)	0.042 (5)	0.068 (5)	0.006 (4)	−0.009 (5)	0.001 (4)
C9	0.059 (5)	0.065 (6)	0.046 (4)	0.013 (4)	0.010 (4)	−0.004 (4)
C10	0.039 (4)	0.043 (4)	0.050 (5)	0.009 (4)	0.004 (4)	0.008 (4)
C11	0.055 (5)	0.061 (6)	0.062 (5)	−0.003 (4)	0.011 (4)	−0.004 (4)
C12	0.076 (6)	0.056 (5)	0.086 (7)	−0.007 (5)	0.015 (6)	0.002 (5)
C13	0.073 (6)	0.097 (10)	0.075 (6)	−0.004 (6)	0.021 (5)	0.022 (6)
C14	0.062 (6)	0.085 (7)	0.060 (6)	0.004 (5)	0.012 (5)	0.001 (5)
C15	0.047 (4)	0.059 (5)	0.046 (5)	0.008 (4)	0.004 (4)	0.002 (4)
C16	0.066 (6)	0.065 (6)	0.063 (5)	0.010 (5)	0.001 (5)	−0.021 (5)
C17	0.070 (6)	0.053 (6)	0.078 (6)	−0.004 (4)	0.001 (5)	−0.011 (4)
C18	0.072 (6)	0.039 (4)	0.073 (6)	−0.005 (4)	0.012 (5)	−0.004 (5)

### Dichloridobis(quinoline N-oxide-κO)zinc(II) (I) Geometric parameters (Å, º)

**Table:**

Zn1—Cl1	2.215 (2)	C7—H7	0.9300
Zn1—Cl2	2.211 (2)	C7—C8	1.350 (11)
Zn1—O1	1.959 (5)	C8—H8	0.9300
Zn1—O2	1.991 (4)	C8—C9	1.393 (10)
O1—N1	1.351 (7)	C9—H9	0.9300
O2—N2	1.339 (6)	C10—C11	1.398 (10)
N1—C1	1.389 (8)	C10—C15	1.412 (10)
N1—C9	1.316 (9)	C11—H11	0.9300
N2—C10	1.385 (8)	C11—C12	1.366 (10)
N2—C18	1.324 (9)	C12—H12	0.9300
C1—C2	1.395 (9)	C12—C13	1.421 (12)
C1—C6	1.403 (9)	C13—H13	0.9300
C2—H2	0.9300	C13—C14	1.344 (12)
C2—C3	1.373 (10)	C14—H14	0.9300
C3—H3	0.9300	C14—C15	1.399 (10)
C3—C4	1.390 (11)	C15—C16	1.420 (11)
C4—H4	0.9300	C16—H16	0.9300
C4—C5	1.350 (12)	C16—C17	1.362 (11)
C5—H5	0.9300	C17—H17	0.9300
C5—C6	1.417 (10)	C17—C18	1.373 (10)
C6—C7	1.412 (10)	C18—H18	0.9300
			
Cl2—Zn1—Cl1	117.80 (9)	C8—C7—H7	119.9
O1—Zn1—Cl1	113.30 (15)	C7—C8—H8	120.2
O1—Zn1—Cl2	104.40 (18)	C7—C8—C9	119.7 (7)
O1—Zn1—O2	99.4 (2)	C9—C8—H8	120.2
O2—Zn1—Cl1	108.81 (16)	N1—C9—C8	121.1 (7)
O2—Zn1—Cl2	111.57 (16)	N1—C9—H9	119.4
N1—O1—Zn1	121.7 (4)	C8—C9—H9	119.4
N2—O2—Zn1	124.0 (4)	N2—C10—C11	119.6 (7)
O1—N1—C1	117.0 (6)	N2—C10—C15	118.5 (7)
C9—N1—O1	121.2 (6)	C11—C10—C15	121.9 (7)
C9—N1—C1	121.8 (6)	C10—C11—H11	120.8
O2—N2—C10	118.8 (6)	C12—C11—C10	118.4 (8)
C18—N2—O2	120.2 (6)	C12—C11—H11	120.8
C18—N2—C10	120.9 (6)	C11—C12—H12	119.9
N1—C1—C2	120.1 (7)	C11—C12—C13	120.2 (9)
N1—C1—C6	118.3 (7)	C13—C12—H12	119.9
C2—C1—C6	121.6 (7)	C12—C13—H13	119.6
C1—C2—H2	120.5	C14—C13—C12	120.9 (9)
C3—C2—C1	118.9 (7)	C14—C13—H13	119.6
C3—C2—H2	120.5	C13—C14—H14	119.5
C2—C3—H3	119.8	C13—C14—C15	121.0 (9)
C2—C3—C4	120.4 (8)	C15—C14—H14	119.5
C4—C3—H3	119.8	C10—C15—C16	119.2 (7)
C3—C4—H4	119.5	C14—C15—C10	117.6 (8)
C5—C4—C3	121.0 (8)	C14—C15—C16	123.2 (8)
C5—C4—H4	119.5	C15—C16—H16	120.6
C4—C5—H5	119.6	C17—C16—C15	118.8 (7)
C4—C5—C6	120.9 (8)	C17—C16—H16	120.6
C6—C5—H5	119.6	C16—C17—H17	119.8
C1—C6—C5	117.2 (7)	C16—C17—C18	120.3 (8)
C1—C6—C7	118.8 (7)	C18—C17—H17	119.8
C7—C6—C5	124.0 (7)	N2—C18—C17	122.1 (8)
C6—C7—H7	119.9	N2—C18—H18	118.9
C8—C7—C6	120.3 (7)	C17—C18—H18	118.9
			
Zn1—O1—N1—C1	127.4 (5)	C4—C5—C6—C1	0.5 (11)
Zn1—O1—N1—C9	−54.6 (8)	C4—C5—C6—C7	−178.7 (8)
Zn1—O2—N2—C10	−94.8 (6)	C5—C6—C7—C8	179.3 (7)
Zn1—O2—N2—C18	88.5 (7)	C6—C1—C2—C3	0.0 (12)
O1—N1—C1—C2	0.3 (10)	C6—C7—C8—C9	1.6 (12)
O1—N1—C1—C6	−179.2 (6)	C7—C8—C9—N1	−1.1 (12)
O1—N1—C9—C8	−179.1 (6)	C9—N1—C1—C2	−177.7 (7)
O2—N2—C10—C11	5.0 (9)	C9—N1—C1—C6	2.8 (10)
O2—N2—C10—C15	−173.8 (6)	C10—N2—C18—C17	−2.7 (11)
O2—N2—C18—C17	174.0 (6)	C10—C11—C12—C13	1.4 (12)
N1—C1—C2—C3	−179.5 (6)	C10—C15—C16—C17	−0.7 (11)
N1—C1—C6—C5	178.5 (6)	C11—C10—C15—C14	0.1 (11)
N1—C1—C6—C7	−2.2 (10)	C11—C10—C15—C16	180.0 (7)
N2—C10—C11—C12	−179.7 (7)	C11—C12—C13—C14	−1.2 (14)
N2—C10—C15—C14	179.0 (6)	C12—C13—C14—C15	0.4 (14)
N2—C10—C15—C16	−1.2 (10)	C13—C14—C15—C10	0.1 (13)
C1—N1—C9—C8	−1.1 (11)	C13—C14—C15—C16	−179.7 (8)
C1—C2—C3—C4	1.4 (12)	C14—C15—C16—C17	179.1 (8)
C1—C6—C7—C8	0.1 (11)	C15—C10—C11—C12	−0.9 (11)
C2—C1—C6—C5	−1.0 (11)	C15—C16—C17—C18	1.0 (12)
C2—C1—C6—C7	178.3 (7)	C16—C17—C18—N2	0.7 (13)
C2—C3—C4—C5	−1.9 (13)	C18—N2—C10—C11	−178.3 (7)
C3—C4—C5—C6	0.9 (13)	C18—N2—C10—C15	2.9 (10)

### Dibromidobis(quinoline N-oxide-κO)zinc(II) (II) Crystal data

**Table:**

[ZnBr_2_(C_9_H_7_NO)_2_]	*F*(000) = 1008
*M**_r_* = 515.50	*D*_x_ = 1.837 Mg m^−^^3^
Monoclinic, *P*2_1_/*c*	Mo *K*α radiation, λ = 0.71073 Å
*a* = 16.3922 (11) Å	Cell parameters from 1219 reflections
*b* = 7.3527 (6) Å	θ = 2.6–22.0°
*c* = 15.5809 (10) Å	µ = 5.62 mm^−^^1^
β = 97.113 (6)°	*T* = 298 K
*V* = 1863.5 (2) Å^3^	Irregular, clear colourless
*Z* = 4	0.15 × 0.08 × 0.03 mm

### Dibromidobis(quinoline N-oxide-κO)zinc(II) (II) Data collection

**Table:**

XtaLAB Mini (ROW) diffractometer	3415 independent reflections
Radiation source: fine-focus sealed X-ray tube, Rigaku (Mo) X-ray Source	2095 reflections with *I* > 2σ(*I*)
Graphite monochromator	*R*_int_ = 0.043
ω scans	θ_max_ = 25.4°, θ_min_ = 2.5°
Absorption correction: multi-scan (CrysAlisPro; Rigaku OD, 2019)	*h* = −16→19
*T*_min_ = 0.833, *T*_max_ = 1.000	*k* = −8→8
7207 measured reflections	*l* = −18→18

### Dibromidobis(quinoline N-oxide-κO)zinc(II) (II) Refinement

**Table:**

Refinement on *F*^2^	Primary atom site location: dual
Least-squares matrix: full	Hydrogen site location: inferred from neighbouring sites
*R*[*F*^2^ > 2σ(*F*^2^)] = 0.042	H-atom parameters constrained
*wR*(*F*^2^) = 0.090	*w* = 1/[σ^2^(*F*_o_^2^) + (0.0258*P*)^2^] where *P* = (*F*_o_^2^ + 2*F*_c_^2^)/3
*S* = 1.01	(Δ/σ)_max_ < 0.001
3415 reflections	Δρ_max_ = 0.55 e Å^−^^3^
226 parameters	Δρ_min_ = −0.35 e Å^−^^3^
0 restraints	

### Dibromidobis(quinoline N-oxide-κO)zinc(II) (II) Special details

**Table:**

Geometry. All esds (except the esd in the dihedral angle between two l.s. planes) are estimated using the full covariance matrix. The cell esds are taken into account individually in the estimation of esds in distances, angles and torsion angles; correlations between esds in cell parameters are only used when they are defined by crystal symmetry. An approximate (isotropic) treatment of cell esds is used for estimating esds involving l.s. planes.

### Dibromidobis(quinoline N-oxide-κO)zinc(II) (II) Fractional atomic coordinates and isotropic or equivalent isotropic displacement parameters (Å^2^)

**Table:**

	*x*	*y*	*z*	*U*_iso_*/*U*_eq_	
Zn1	0.25508 (4)	0.26213 (9)	0.37264 (4)	0.0514 (2)	
Br2	0.22409 (4)	−0.03623 (9)	0.41131 (4)	0.0695 (2)	
Br1	0.26119 (4)	0.35514 (10)	0.22878 (4)	0.0698 (2)	
O1	0.3616 (2)	0.3196 (6)	0.4411 (2)	0.0662 (11)	
O2	0.1778 (2)	0.4332 (5)	0.4197 (2)	0.0597 (10)	
N2	0.1157 (3)	0.3586 (5)	0.4557 (3)	0.0445 (11)	
N1	0.4115 (3)	0.4386 (7)	0.4079 (3)	0.0543 (12)	
C10	0.0394 (3)	0.3446 (7)	0.4065 (3)	0.0415 (12)	
C1	0.4897 (3)	0.3784 (8)	0.3969 (3)	0.0466 (14)	
C15	−0.0264 (3)	0.2713 (7)	0.4450 (3)	0.0468 (13)	
C16	−0.0121 (4)	0.2148 (7)	0.5319 (3)	0.0550 (15)	
H16	−0.054979	0.167100	0.558720	0.066*	
C18	0.1273 (3)	0.3032 (7)	0.5371 (3)	0.0526 (15)	
H18	0.179191	0.313686	0.568455	0.063*	
C17	0.0635 (4)	0.2296 (8)	0.5765 (3)	0.0561 (15)	
H17	0.072791	0.190483	0.633590	0.067*	
C11	0.0284 (4)	0.4086 (8)	0.3210 (3)	0.0571 (16)	
H11	0.071759	0.460347	0.296363	0.069*	
C6	0.5437 (4)	0.5023 (9)	0.3641 (3)	0.0592 (16)	
C2	0.5136 (4)	0.2028 (9)	0.4187 (3)	0.0626 (17)	
H2	0.476904	0.122160	0.439392	0.075*	
C14	−0.1031 (4)	0.2572 (8)	0.3940 (4)	0.0673 (17)	
H14	−0.147290	0.206267	0.417522	0.081*	
C13	−0.1135 (4)	0.3168 (9)	0.3113 (4)	0.0742 (19)	
H13	−0.164632	0.307127	0.278474	0.089*	
C12	−0.0477 (4)	0.3927 (9)	0.2752 (3)	0.0723 (19)	
H12	−0.055985	0.433696	0.218351	0.087*	
C9	0.3862 (4)	0.6041 (10)	0.3872 (4)	0.0730 (19)	
H9	0.333060	0.639141	0.395028	0.088*	
C7	0.5161 (5)	0.6777 (10)	0.3420 (4)	0.077 (2)	
H7	0.550832	0.760022	0.319363	0.093*	
C3	0.5912 (4)	0.1490 (10)	0.4098 (4)	0.083 (2)	
H3	0.607681	0.030702	0.424267	0.099*	
C8	0.4388 (5)	0.7279 (9)	0.3536 (4)	0.083 (2)	
H8	0.420515	0.844968	0.339148	0.099*	
C5	0.6244 (4)	0.4382 (12)	0.3568 (4)	0.085 (2)	
H5	0.662411	0.515411	0.336046	0.102*	
C4	0.6460 (5)	0.2678 (14)	0.3794 (5)	0.095 (3)	
H4	0.699190	0.228696	0.374594	0.114*	

### Dibromidobis(quinoline N-oxide-κO)zinc(II) (II) Atomic displacement parameters (Å^2^)

**Table:**

	*U*^11^	*U*^22^	*U*^33^	*U*^12^	*U*^13^	*U*^23^
Zn1	0.0345 (4)	0.0688 (5)	0.0519 (4)	−0.0003 (3)	0.0089 (3)	−0.0017 (3)
Br2	0.0598 (4)	0.0637 (4)	0.0855 (5)	0.0040 (3)	0.0113 (3)	0.0048 (4)
Br1	0.0663 (4)	0.0960 (5)	0.0476 (3)	−0.0052 (4)	0.0090 (3)	−0.0030 (4)
O1	0.037 (2)	0.102 (3)	0.059 (2)	−0.016 (2)	0.0020 (18)	0.010 (2)
O2	0.046 (2)	0.055 (2)	0.083 (3)	−0.005 (2)	0.0278 (19)	0.002 (2)
N2	0.040 (3)	0.042 (3)	0.054 (3)	0.007 (2)	0.014 (2)	−0.005 (2)
N1	0.043 (3)	0.072 (4)	0.045 (3)	−0.005 (3)	−0.007 (2)	−0.012 (3)
C10	0.043 (3)	0.039 (3)	0.043 (3)	0.007 (3)	0.011 (2)	−0.006 (3)
C1	0.039 (3)	0.062 (4)	0.037 (3)	−0.005 (3)	−0.002 (2)	−0.011 (3)
C15	0.041 (3)	0.048 (3)	0.054 (3)	0.003 (3)	0.015 (3)	−0.003 (3)
C16	0.051 (4)	0.060 (4)	0.057 (4)	−0.001 (3)	0.020 (3)	0.006 (3)
C18	0.053 (4)	0.056 (4)	0.047 (3)	0.009 (3)	−0.003 (3)	−0.004 (3)
C17	0.063 (4)	0.062 (4)	0.046 (3)	0.007 (3)	0.017 (3)	0.008 (3)
C11	0.064 (4)	0.063 (4)	0.046 (3)	0.008 (3)	0.015 (3)	−0.003 (3)
C6	0.051 (4)	0.076 (5)	0.049 (3)	−0.016 (4)	0.001 (3)	−0.015 (3)
C2	0.053 (4)	0.072 (5)	0.059 (4)	−0.003 (3)	−0.007 (3)	−0.003 (3)
C14	0.042 (4)	0.079 (5)	0.081 (5)	−0.008 (3)	0.008 (3)	0.000 (4)
C13	0.055 (4)	0.092 (5)	0.072 (4)	0.002 (4)	−0.007 (3)	−0.006 (4)
C12	0.085 (5)	0.094 (5)	0.037 (3)	0.016 (4)	0.002 (3)	−0.002 (3)
C9	0.052 (4)	0.088 (5)	0.075 (4)	0.009 (4)	−0.010 (3)	−0.028 (4)
C7	0.086 (6)	0.074 (5)	0.071 (4)	−0.029 (4)	0.002 (4)	−0.003 (4)
C3	0.064 (5)	0.079 (5)	0.101 (5)	0.010 (4)	−0.007 (4)	−0.016 (4)
C8	0.098 (6)	0.053 (4)	0.087 (5)	−0.003 (5)	−0.028 (5)	0.000 (4)
C5	0.056 (5)	0.122 (7)	0.079 (5)	−0.035 (5)	0.022 (4)	−0.023 (5)
C4	0.050 (5)	0.130 (7)	0.104 (6)	0.006 (5)	0.008 (4)	−0.029 (6)

### Dibromidobis(quinoline N-oxide-κO)zinc(II) (II) Geometric parameters (Å, º)

**Table:**

Zn1—Br2	2.3472 (10)	C11—H11	0.9300
Zn1—Br1	2.3575 (8)	C11—C12	1.364 (8)
Zn1—O1	1.975 (3)	C6—C7	1.395 (8)
Zn1—O2	1.989 (4)	C6—C5	1.422 (9)
O1—N1	1.345 (5)	C2—H2	0.9300
O2—N2	1.339 (5)	C2—C3	1.356 (8)
N2—C10	1.388 (6)	C14—H14	0.9300
N2—C18	1.323 (6)	C14—C13	1.352 (8)
N1—C1	1.386 (6)	C13—H13	0.9300
N1—C9	1.313 (7)	C13—C12	1.392 (8)
C10—C15	1.406 (7)	C12—H12	0.9300
C10—C11	1.402 (7)	C9—H9	0.9300
C1—C6	1.410 (7)	C9—C8	1.400 (9)
C1—C2	1.380 (7)	C7—H7	0.9300
C15—C16	1.408 (7)	C7—C8	1.354 (9)
C15—C14	1.405 (7)	C3—H3	0.9300
C16—H16	0.9300	C3—C4	1.378 (10)
C16—C17	1.346 (7)	C8—H8	0.9300
C18—H18	0.9300	C5—H5	0.9300
C18—C17	1.387 (7)	C5—C4	1.338 (9)
C17—H17	0.9300	C4—H4	0.9300
			
Br2—Zn1—Br1	123.45 (4)	C12—C11—H11	121.0
O1—Zn1—Br2	105.44 (12)	C1—C6—C5	116.5 (6)
O1—Zn1—Br1	108.21 (11)	C7—C6—C1	119.2 (6)
O1—Zn1—O2	103.10 (16)	C7—C6—C5	124.2 (7)
O2—Zn1—Br2	109.17 (11)	C1—C2—H2	120.4
O2—Zn1—Br1	105.72 (11)	C3—C2—C1	119.3 (6)
N1—O1—Zn1	118.1 (3)	C3—C2—H2	120.4
N2—O2—Zn1	116.6 (3)	C15—C14—H14	119.6
O2—N2—C10	118.5 (4)	C13—C14—C15	120.8 (6)
C18—N2—O2	120.1 (4)	C13—C14—H14	119.6
C18—N2—C10	121.4 (5)	C14—C13—H13	119.9
O1—N1—C1	117.1 (5)	C14—C13—C12	120.2 (6)
C9—N1—O1	120.5 (5)	C12—C13—H13	119.9
C9—N1—C1	122.4 (6)	C11—C12—C13	121.8 (6)
N2—C10—C15	118.6 (5)	C11—C12—H12	119.1
N2—C10—C11	120.1 (5)	C13—C12—H12	119.1
C11—C10—C15	121.3 (5)	N1—C9—H9	119.9
N1—C1—C6	118.0 (6)	N1—C9—C8	120.2 (6)
C2—C1—N1	120.5 (5)	C8—C9—H9	119.9
C2—C1—C6	121.5 (6)	C6—C7—H7	120.0
C10—C15—C16	118.6 (5)	C8—C7—C6	119.9 (7)
C14—C15—C10	117.9 (5)	C8—C7—H7	120.0
C14—C15—C16	123.6 (5)	C2—C3—H3	119.7
C15—C16—H16	119.8	C2—C3—C4	120.7 (7)
C17—C16—C15	120.3 (5)	C4—C3—H3	119.7
C17—C16—H16	119.8	C9—C8—H8	119.9
N2—C18—H18	119.4	C7—C8—C9	120.2 (7)
N2—C18—C17	121.1 (5)	C7—C8—H8	119.9
C17—C18—H18	119.4	C6—C5—H5	119.7
C16—C17—C18	120.0 (5)	C4—C5—C6	120.5 (7)
C16—C17—H17	120.0	C4—C5—H5	119.7
C18—C17—H17	120.0	C3—C4—H4	119.3
C10—C11—H11	121.0	C5—C4—C3	121.5 (7)
C12—C11—C10	118.0 (6)	C5—C4—H4	119.3
			
Zn1—O1—N1—C1	−122.3 (4)	C1—C6—C7—C8	1.1 (9)
Zn1—O1—N1—C9	57.8 (6)	C1—C6—C5—C4	0.7 (9)
Zn1—O2—N2—C10	−97.8 (4)	C1—C2—C3—C4	0.2 (9)
Zn1—O2—N2—C18	83.4 (5)	C15—C10—C11—C12	−2.0 (8)
O1—N1—C1—C6	−178.5 (4)	C15—C16—C17—C18	0.9 (9)
O1—N1—C1—C2	0.7 (7)	C15—C14—C13—C12	0.3 (10)
O1—N1—C9—C8	179.3 (5)	C16—C15—C14—C13	178.8 (6)
O2—N2—C10—C15	−178.1 (4)	C18—N2—C10—C15	0.6 (7)
O2—N2—C10—C11	−0.3 (7)	C18—N2—C10—C11	178.4 (5)
O2—N2—C18—C17	178.4 (5)	C11—C10—C15—C16	−177.9 (5)
N2—C10—C15—C16	−0.2 (7)	C11—C10—C15—C14	2.6 (8)
N2—C10—C15—C14	−179.7 (5)	C6—C1—C2—C3	1.1 (8)
N2—C10—C11—C12	−179.7 (5)	C6—C7—C8—C9	−0.3 (10)
N2—C18—C17—C16	−0.5 (8)	C6—C5—C4—C3	0.5 (11)
N1—C1—C6—C7	−1.6 (7)	C2—C1—C6—C7	179.2 (5)
N1—C1—C6—C5	177.7 (5)	C2—C1—C6—C5	−1.5 (8)
N1—C1—C2—C3	−178.1 (5)	C2—C3—C4—C5	−1.0 (11)
N1—C9—C8—C7	0.1 (10)	C14—C15—C16—C17	178.9 (6)
C10—N2—C18—C17	−0.3 (8)	C14—C13—C12—C11	0.4 (10)
C10—C15—C16—C17	−0.6 (8)	C9—N1—C1—C6	1.4 (7)
C10—C15—C14—C13	−1.7 (9)	C9—N1—C1—C2	−179.4 (5)
C10—C11—C12—C13	0.5 (9)	C7—C6—C5—C4	179.9 (6)
C1—N1—C9—C8	−0.6 (8)	C5—C6—C7—C8	−178.1 (6)

### Diiodidodobis(quinoline N-oxide-κO)zinc(II) (III) Crystal data

**Table:**

[ZnI_2_(C_9_H_7_NO)_2_]	*F*(000) = 1152
*M**_r_* = 609.48	*D*_x_ = 2.019 Mg m^−^^3^
Monoclinic, *P*2_1_/*c*	Mo *K*α radiation, λ = 0.71073 Å
*a* = 16.7231 (7) Å	Cell parameters from 3422 reflections
*b* = 7.6155 (4) Å	θ = 2.6–24.1°
*c* = 15.8689 (7) Å	µ = 4.32 mm^−^^1^
β = 97.192 (4)°	*T* = 297 K
*V* = 2005.08 (16) Å^3^	Block, clear colourless
*Z* = 4	0.3 × 0.3 × 0.3 mm

### Diiodidodobis(quinoline N-oxide-κO)zinc(II) (III) Data collection

**Table:**

Rigaku XtaLAB mini diffractometer	2748 reflections with *I* > 2σ(*I*)
ω scans	*R*_int_ = 0.032
Absorption correction: multi-scan (CrysAlisPro; Rigaku OD, 2019)	θ_max_ = 25.4°, θ_min_ = 2.5°
*T*_min_ = 0.896, *T*_max_ = 1.000	*h* = −20→20
11510 measured reflections	*k* = −8→9
3668 independent reflections	*l* = −19→19

### Diiodidodobis(quinoline N-oxide-κO)zinc(II) (III) Refinement

**Table:**

Refinement on *F*^2^	Hydrogen site location: inferred from neighbouring sites
Least-squares matrix: full	H-atom parameters constrained
*R*[*F*^2^ > 2σ(*F*^2^)] = 0.035	*w* = 1/[σ^2^(*F*_o_^2^) + (0.0249*P*)^2^ + 3.8317*P*] where *P* = (*F*_o_^2^ + 2*F*_c_^2^)/3
*wR*(*F*^2^) = 0.085	(Δ/σ)_max_ < 0.001
*S* = 1.07	Δρ_max_ = 0.80 e Å^−^^3^
3668 reflections	Δρ_min_ = −0.81 e Å^−^^3^
227 parameters	Extinction correction: SHELXL-2018/1 (Sheldrick 2015a), Fc^*^=kFc[1+0.001xFc^2^λ^3^/sin(2θ)]^-1/4^
0 restraints	Extinction coefficient: 0.00071 (11)

### Diiodidodobis(quinoline N-oxide-κO)zinc(II) (III) Special details

**Table:**

Geometry. All esds (except the esd in the dihedral angle between two l.s. planes) are estimated using the full covariance matrix. The cell esds are taken into account individually in the estimation of esds in distances, angles and torsion angles; correlations between esds in cell parameters are only used when they are defined by crystal symmetry. An approximate (isotropic) treatment of cell esds is used for estimating esds involving l.s. planes.

### Diiodidodobis(quinoline N-oxide-κO)zinc(II) (III) Fractional atomic coordinates and isotropic or equivalent isotropic displacement parameters (Å^2^)

**Table:**

	*x*	*y*	*z*	*U*_iso_*/*U*_eq_	
I1	0.26214 (2)	0.37499 (6)	0.22453 (2)	0.07021 (17)	
I2	0.22463 (3)	−0.02923 (6)	0.41988 (3)	0.07442 (17)	
Zn1	0.25578 (3)	0.28426 (10)	0.37845 (4)	0.0553 (2)	
O1	0.3601 (2)	0.3426 (7)	0.4449 (3)	0.0780 (13)	
O2	0.1777 (2)	0.4466 (5)	0.4234 (3)	0.0627 (10)	
N1	0.4094 (3)	0.4544 (7)	0.4109 (3)	0.0592 (12)	
N2	0.1160 (3)	0.3728 (6)	0.4566 (3)	0.0509 (11)	
C1	0.4847 (3)	0.3925 (8)	0.3984 (3)	0.0545 (14)	
C2	0.5069 (4)	0.2175 (9)	0.4180 (4)	0.0707 (17)	
H2	0.470599	0.140389	0.438341	0.085*	
C3	0.5817 (5)	0.1635 (11)	0.4068 (5)	0.095 (2)	
H3	0.596812	0.048098	0.419687	0.114*	
C4	0.6360 (5)	0.2760 (13)	0.3768 (6)	0.105 (3)	
H4	0.687500	0.235837	0.370683	0.126*	
C5	0.6159 (4)	0.4433 (12)	0.3560 (5)	0.088 (2)	
H5	0.653406	0.516920	0.335438	0.106*	
C6	0.5378 (4)	0.5077 (9)	0.3652 (4)	0.0641 (16)	
C7	0.5127 (5)	0.6797 (10)	0.3444 (5)	0.082 (2)	
H7	0.547198	0.757244	0.321637	0.099*	
C8	0.4374 (5)	0.7327 (10)	0.3576 (5)	0.085 (2)	
H8	0.419938	0.846144	0.343511	0.102*	
C9	0.3871 (4)	0.6157 (10)	0.3923 (4)	0.0758 (19)	
H9	0.336222	0.652818	0.402601	0.091*	
C10	0.0431 (3)	0.3575 (7)	0.4054 (3)	0.0487 (12)	
C11	0.0343 (4)	0.4227 (8)	0.3219 (4)	0.0638 (16)	
H11	0.077050	0.475911	0.299604	0.077*	
C12	−0.0388 (5)	0.4051 (10)	0.2751 (4)	0.081 (2)	
H12	−0.046101	0.448351	0.219880	0.098*	
C13	−0.1039 (4)	0.3237 (11)	0.3073 (5)	0.088 (2)	
H13	−0.153042	0.311918	0.273148	0.106*	
C14	−0.0955 (4)	0.2624 (9)	0.3879 (5)	0.0755 (19)	
H14	−0.138783	0.208570	0.408920	0.091*	
C15	−0.0218 (3)	0.2796 (7)	0.4398 (4)	0.0534 (13)	
C16	−0.0094 (4)	0.2205 (8)	0.5249 (4)	0.0632 (16)	
H16	−0.051513	0.168071	0.548760	0.076*	
C17	0.0633 (4)	0.2398 (9)	0.5717 (4)	0.0666 (17)	
H17	0.071247	0.201324	0.627730	0.080*	
C18	0.1257 (4)	0.3171 (8)	0.5358 (4)	0.0589 (15)	
H18	0.175593	0.330122	0.568251	0.071*	

### Diiodidodobis(quinoline N-oxide-κO)zinc(II) (III) Atomic displacement parameters (Å^2^)

**Table:**

	*U*^11^	*U*^22^	*U*^33^	*U*^12^	*U*^13^	*U*^23^
I1	0.0640 (3)	0.0961 (4)	0.0508 (2)	−0.0078 (2)	0.00801 (19)	−0.0018 (2)
I2	0.0652 (3)	0.0668 (3)	0.0928 (3)	0.0106 (2)	0.0157 (2)	0.0090 (2)
Zn1	0.0377 (3)	0.0742 (5)	0.0542 (4)	−0.0006 (3)	0.0070 (3)	−0.0005 (3)
O1	0.048 (2)	0.122 (4)	0.063 (3)	−0.014 (2)	0.002 (2)	0.006 (3)
O2	0.055 (2)	0.059 (3)	0.078 (3)	−0.0007 (19)	0.024 (2)	0.003 (2)
N1	0.043 (3)	0.079 (4)	0.052 (3)	−0.001 (3)	−0.003 (2)	−0.012 (3)
N2	0.047 (2)	0.052 (3)	0.057 (3)	0.007 (2)	0.016 (2)	−0.001 (2)
C1	0.042 (3)	0.075 (4)	0.044 (3)	−0.001 (3)	−0.005 (2)	−0.014 (3)
C2	0.066 (4)	0.069 (5)	0.073 (4)	−0.001 (3)	−0.007 (3)	−0.004 (3)
C3	0.073 (5)	0.086 (6)	0.120 (7)	0.013 (4)	−0.009 (5)	−0.026 (5)
C4	0.064 (5)	0.114 (7)	0.136 (8)	0.012 (5)	0.011 (5)	−0.054 (6)
C5	0.061 (4)	0.110 (7)	0.097 (6)	−0.016 (4)	0.021 (4)	−0.026 (5)
C6	0.053 (3)	0.073 (5)	0.066 (4)	−0.011 (3)	0.006 (3)	−0.017 (3)
C7	0.083 (5)	0.076 (5)	0.086 (5)	−0.022 (4)	0.001 (4)	−0.008 (4)
C8	0.087 (5)	0.065 (5)	0.095 (5)	0.004 (4)	−0.018 (4)	−0.013 (4)
C9	0.063 (4)	0.084 (5)	0.076 (4)	0.008 (4)	−0.011 (4)	−0.024 (4)
C10	0.051 (3)	0.047 (3)	0.049 (3)	0.007 (2)	0.013 (3)	0.000 (2)
C11	0.070 (4)	0.071 (4)	0.051 (3)	0.005 (3)	0.012 (3)	0.003 (3)
C12	0.092 (5)	0.099 (6)	0.052 (4)	0.014 (4)	0.002 (4)	−0.001 (4)
C13	0.065 (4)	0.110 (6)	0.085 (5)	0.005 (4)	−0.015 (4)	−0.006 (5)
C14	0.056 (4)	0.083 (5)	0.086 (5)	−0.008 (3)	0.004 (4)	−0.010 (4)
C15	0.050 (3)	0.053 (3)	0.058 (3)	0.004 (3)	0.011 (3)	−0.003 (3)
C16	0.062 (4)	0.060 (4)	0.071 (4)	0.005 (3)	0.025 (3)	0.013 (3)
C17	0.067 (4)	0.080 (5)	0.056 (4)	0.014 (3)	0.016 (3)	0.011 (3)
C18	0.056 (3)	0.069 (4)	0.051 (3)	0.009 (3)	0.005 (3)	0.000 (3)

### Diiodidodobis(quinoline N-oxide-κO)zinc(II) (III) Geometric parameters (Å, º)

**Table:**

I1—Zn1	2.5534 (8)	C7—H7	0.9300
I2—Zn1	2.5473 (9)	C7—C8	1.363 (10)
Zn1—O1	1.973 (4)	C8—H8	0.9300
Zn1—O2	1.994 (4)	C8—C9	1.386 (10)
O1—N1	1.345 (6)	C9—H9	0.9300
O2—N2	1.339 (5)	C10—C11	1.405 (8)
N1—C1	1.381 (7)	C10—C15	1.405 (7)
N1—C9	1.307 (8)	C11—H11	0.9300
N2—C10	1.383 (7)	C11—C12	1.356 (9)
N2—C18	1.317 (7)	C12—H12	0.9300
C1—C2	1.408 (9)	C12—C13	1.404 (10)
C1—C6	1.397 (8)	C13—H13	0.9300
C2—H2	0.9300	C13—C14	1.352 (10)
C2—C3	1.350 (9)	C14—H14	0.9300
C3—H3	0.9300	C14—C15	1.400 (8)
C3—C4	1.377 (12)	C15—C16	1.414 (8)
C4—H4	0.9300	C16—H16	0.9300
C4—C5	1.348 (12)	C16—C17	1.350 (8)
C5—H5	0.9300	C17—H17	0.9300
C5—C6	1.420 (9)	C17—C18	1.382 (8)
C6—C7	1.402 (10)	C18—H18	0.9300
			
I2—Zn1—I1	122.33 (3)	C8—C7—H7	120.2
O1—Zn1—I1	108.10 (13)	C7—C8—H8	120.4
O1—Zn1—I2	105.60 (15)	C7—C8—C9	119.3 (7)
O1—Zn1—O2	104.13 (19)	C9—C8—H8	120.4
O2—Zn1—I1	106.36 (12)	N1—C9—C8	121.6 (7)
O2—Zn1—I2	108.93 (12)	N1—C9—H9	119.2
N1—O1—Zn1	118.3 (3)	C8—C9—H9	119.2
N2—O2—Zn1	116.9 (3)	N2—C10—C11	120.3 (5)
O1—N1—C1	117.2 (5)	N2—C10—C15	118.3 (5)
C9—N1—O1	120.9 (5)	C15—C10—C11	121.4 (5)
C9—N1—C1	121.9 (6)	C10—C11—H11	121.2
O2—N2—C10	118.0 (4)	C12—C11—C10	117.6 (6)
C18—N2—O2	120.2 (5)	C12—C11—H11	121.2
C18—N2—C10	121.8 (5)	C11—C12—H12	118.9
N1—C1—C2	120.7 (6)	C11—C12—C13	122.1 (6)
N1—C1—C6	118.3 (6)	C13—C12—H12	118.9
C6—C1—C2	121.0 (6)	C12—C13—H13	119.9
C1—C2—H2	120.6	C14—C13—C12	120.2 (7)
C3—C2—C1	118.8 (7)	C14—C13—H13	119.9
C3—C2—H2	120.6	C13—C14—H14	119.8
C2—C3—H3	119.4	C13—C14—C15	120.3 (7)
C2—C3—C4	121.3 (8)	C15—C14—H14	119.8
C4—C3—H3	119.4	C10—C15—C16	118.5 (5)
C3—C4—H4	119.4	C14—C15—C10	118.4 (5)
C5—C4—C3	121.2 (8)	C14—C15—C16	123.0 (6)
C5—C4—H4	119.4	C15—C16—H16	119.9
C4—C5—H5	119.8	C17—C16—C15	120.3 (6)
C4—C5—C6	120.4 (8)	C17—C16—H16	119.9
C6—C5—H5	119.8	C16—C17—H17	120.2
C1—C6—C5	117.3 (7)	C16—C17—C18	119.6 (6)
C1—C6—C7	119.3 (6)	C18—C17—H17	120.2
C7—C6—C5	123.4 (7)	N2—C18—C17	121.5 (6)
C6—C7—H7	120.2	N2—C18—H18	119.3
C8—C7—C6	119.7 (7)	C17—C18—H18	119.3
			
Zn1—O1—N1—C1	−119.9 (4)	C4—C5—C6—C1	1.6 (10)
Zn1—O1—N1—C9	61.5 (6)	C4—C5—C6—C7	−179.6 (7)
Zn1—O2—N2—C10	−96.9 (5)	C5—C6—C7—C8	−178.0 (7)
Zn1—O2—N2—C18	83.1 (5)	C6—C1—C2—C3	2.1 (9)
O1—N1—C1—C2	2.2 (7)	C6—C7—C8—C9	0.6 (11)
O1—N1—C1—C6	−178.3 (5)	C7—C8—C9—N1	−1.6 (11)
O1—N1—C9—C8	179.7 (5)	C9—N1—C1—C2	−179.2 (6)
O2—N2—C10—C11	−1.7 (7)	C9—N1—C1—C6	0.3 (8)
O2—N2—C10—C15	179.7 (5)	C10—N2—C18—C17	0.3 (9)
O2—N2—C18—C17	−179.7 (5)	C10—C11—C12—C13	−0.6 (11)
N1—C1—C2—C3	−178.4 (6)	C10—C15—C16—C17	0.3 (9)
N1—C1—C6—C5	177.6 (5)	C11—C10—C15—C14	2.1 (9)
N1—C1—C6—C7	−1.2 (8)	C11—C10—C15—C16	−178.5 (5)
N2—C10—C11—C12	−179.5 (6)	C11—C12—C13—C14	1.2 (12)
N2—C10—C15—C14	−179.4 (5)	C12—C13—C14—C15	0.0 (12)
N2—C10—C15—C16	0.0 (8)	C13—C14—C15—C10	−1.6 (10)
C1—N1—C9—C8	1.1 (9)	C13—C14—C15—C16	179.1 (7)
C1—C2—C3—C4	−0.1 (11)	C14—C15—C16—C17	179.6 (6)
C1—C6—C7—C8	0.7 (10)	C15—C10—C11—C12	−1.0 (9)
C2—C1—C6—C5	−2.9 (9)	C15—C16—C17—C18	−0.3 (10)
C2—C1—C6—C7	178.3 (6)	C16—C17—C18—N2	0.0 (10)
C2—C3—C4—C5	−1.2 (13)	C18—N2—C10—C11	178.3 (5)
C3—C4—C5—C6	0.4 (13)	C18—N2—C10—C15	−0.2 (8)
